# Getting Started in Biological Pathway Construction and Analysis

**DOI:** 10.1371/journal.pcbi.0040016

**Published:** 2008-02-29

**Authors:** Ganesh A Viswanathan, Jeremy Seto, Sonali Patil, German Nudelman, Stuart C Sealfon

**Affiliations:** Princeton University, United States of America

## Introduction

Life depends on the capacity of individual cells to respond effectively to cues about their changing internal and external environments. Cellular decision making and responses are orchestrated by complex molecular networks consisting of entities such as proteins or RNAs connected by interactions such as activation or synthesis. Information contained in primary databases and in the experimental literature relevant to these networks is so extensive and rapidly growing that it is increasingly difficult to integrate. As an aid to theoretical and experimental research, it is convenient to distill the inferences contained in the experimental literature and databases into knowledgebases that consist of annotated representations of biological pathways.

Pathway building has been performed by individual groups studying a network of interest (e.g., Kitano's group who assembled an immune signaling pathway [1]) as well as by large bioinformatics consortia (e.g., the Reactome Project [2]) and commercial entities (e.g., Ingenuity Systems). Pathway building is the process of identifying and integrating the entities, interactions, and associated annotations, and populating the knowledgebase. Pathway construction can have either a data-driven objective (DDO) or a knowledge-driven objective (KDO). Data-driven pathway construction is used to generate relationship information of genes or proteins identified in a specific experiment such as a microarray study. Knowledge-driven pathway construction entails development of a detailed pathway knowledgebase for particular domains of interest, such as a cell type, disease, or system. To help researchers get their bearings in this field, in the subsequent sections we provide a brief, practical orientation to existing knowledgebases and to the methods of pathway construction and analysis.

## Biological Pathway Construction Workflow

**Figure 1:**
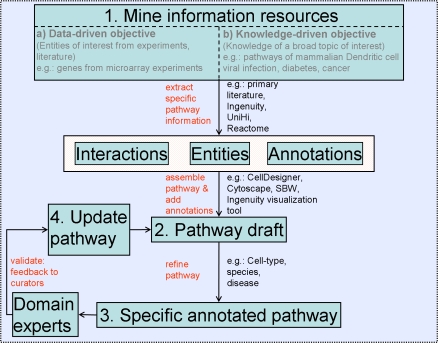
Schematic Illustrating the Biological Pathway Building Process Pathway curators initially mine information (Step 1). The mining process can be initiated by two broad pathway building objectives: (a) DDO wherein a list of genes and/or proteins are obtained by high-throughput experiments such as microarray, mass spectrometry or (b) KDO wherein a broad topic of interest is chosen and then the knowledge concerning this topic is mined from resources such as the primary literature and knowledgebases. Information from the mining process is assembled (Step 2), using pathway building tools, into a pathway, which, following many iterations of feedback from domain experts (Step 3) and refinement (Step 4), leads to the desired specific annotated pathway.

The curation process of a biological pathway entails identifying and structuring content, mining information manually and/or computationally, and assembling a knowledgebase using appropriate software tools. A schematic illustrating the major steps involved in the data-driven and knowledge-driven construction processes is shown in Figure 1. For either DDO or KDO pathway construction, the first step is to mine pertinent information from relevant information sources (discussed in Public and Private Information Sources) about the entities and interactions. The information retrieved is assembled using appropriate formats, information standards, and pathway building tools (discussed in Formats, Standards, and Pathway Building Tools) to obtain a pathway prototype. The pathway is further refined to include context-specific annotations such as species, cell/tissue type, or disease type. The pathway can then be verified by the domain experts and updated by the curators based on appropriate feedback. In the section Illustration of the Pathway Building Process, we describe an example of the KDO approach for building a pathway.

## Public and Private Information Sources

The extension of reductive biology begun with Aristotle's *Parts of Animals* to the molecular realm has defined large numbers of entities and interactions in various cells and organisms. Recent attempts to improve knowledge integration have led to refined classifications of cellular entities, such as Gene Ontology (GO), and to the assembly of structured knowledge repositories. Data repositories, which contain information regarding sequence data, metabolism, signaling, reactions, and interactions are a major source of information for pathway building. A few useful databases are described in Table 1. A comprehensive list of resources can be found at http://www.pathguide.org.

**Table 1:**
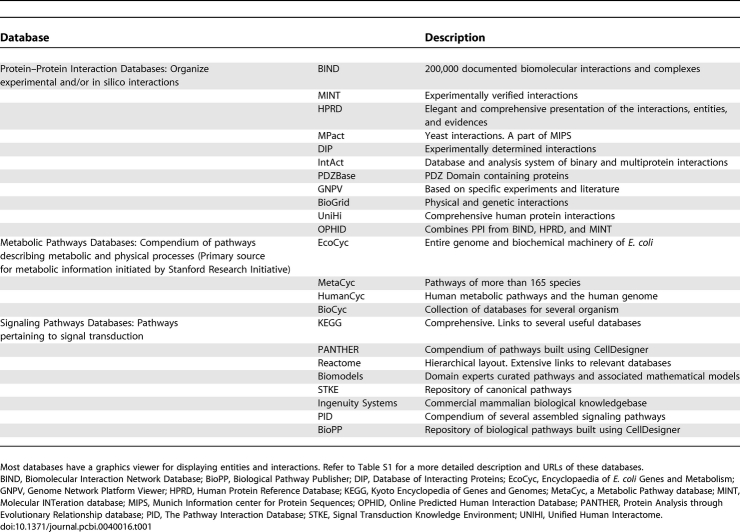
A List of Databases, Classified Based on the Type of Information Represented, Commonly Used during a Biological Pathway Construction

## Formats, Standards, and Pathway Building Tools

Various standard, computer readable, object-oriented formats have been developed to facilitate the organization, storage, exchange, and parsing of pathway knowledgebases and the relevant experimental evidence information. Important pathway and pathway-related formats, which are all XML-based, include Systems Biology Markup Language (SBML), Proteomics Standards Initiative–Molecular Interactions (PSI-MI), and Biological Pathways eXchange (BioPAX) [3]. SBML, which is used mainly for representation of pathways and mathematical models and supported by more than 100 software systems, is currently the best-suited format for mathematical modeling and simulations. PSI-MI is designed for structured representation of experimental evidence information, such as molecular interactions data. The richest format, BioPAX, integrates PSI-MI within a pathway representation format and provides general representation mechanisms that permit storage of additional information, such as mathematical models. However, BioPAX is relatively new, and its features are rapidly evolving, making it a technical challenge to implement. Standards have also been developed for representation of different biological information such as the nomenclature of entities and interactions (e.g., HUGO, Human Genome Organization), and experimental data, (e.g., MIAME, Minimal Information Associated with Microarray Experiments). The ability to extract information automatically and to make inferences is furthered by the use of the controlled vocabularies of established taxonomies and ontologies [4]. GO classifies genes to provide insight into their function and relationships and serves as a model for other biological ontologies. A comprehensive review of biological information standards can be found in [5].

Pathway building tools are required to populate, visualize, and store a pathway. Currently there are various pathway building tools [3] that provide the ability to extract information as well as to support multiple standard formats. Cytoscape, CellDesigner, and JDesigner are graphical environments for constructing pathways that can import/export SBML models for simulation. Cytoscape can also access large databases containing protein and gene interactions with additional support for PSI-MI and BioPAX formats. Pathway Analysis Tools for Integration and Knowledgebase (PATIKA) provides a Web-based interface to public databases, such as Reactome, HPRD, and IntAct through supporting both SBML and BioPAX formats. Its visualization and layout tools facilitate pathway analysis. Reactome displays reactions as pathway diagrams and provides online tools for authoring, curation, and visualization as well as export to SBML and BioPAX formats. Ingenuity pathway analysis tool, a Web-based interface of the Ingenuity Knowledgebase, available by paid subscription, enables users to query molecular interactions, biological functions, and diseases for generating customized pathways and analysis.

## Illustration of the Pathway Building Process

Pathway curation can be either manual or automated. Manual curation provides the most reliable information extraction from the literature. However, the pace of new discovery can make manually populated databases difficult to maintain. In the mining process, use of appropriate keywords increases the chances of identifying the relevant information. Automated text mining through Natural Language Processing reduces the personnel required for recovery of information, but has severe limitations in accuracy. Information in the scientific literature is highly specialized, semantically unpredictable, and often not textual. Agreeing on “facts” is difficult even for expert curators. The present generation of text mining tools is probably most useful as an aid to manual curation.

The efficient mining of information from the plethora of resource databases hinges on the identification of the most useful primary literature and databases for the biological area of interest. This often poses a challenge, as the choice of databases and mining strategies are biological area–specific. We find Reactome, UniHI, and Ingenuity Systems useful and appropriate for many biological areas.

**Figure 2:**
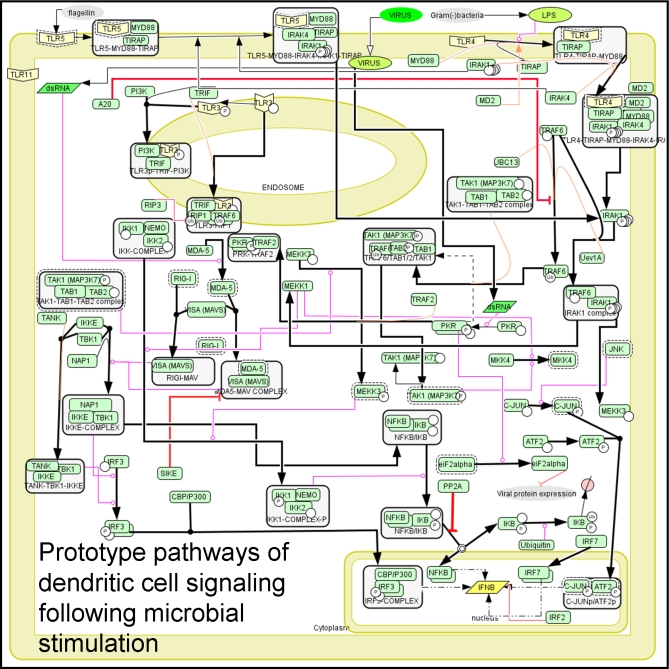
Example of KDO Pathway Assembly: Signal Transduction Pathways Involved during Infection due to Pathogens such as Virus, Bacteria in Mammalian Dendritic Cells Starting from a broad topic of interest—infection in mammalian dendritic cells—using the resources in Table 1, this network of pathways was built.

We provide here an example of assembly of a human dendritic cell signaling pathway involved in responding to microbes, assembled in CellDesigner, built using a KDO-based information mining approach. A snapshot of the pathway is in Figure 2. We extracted information such as TLRs, TRIF, MyD88, RIGI, IRF3, and IFNβ predominantly from primary literature and comprehensive review papers obtained from databases such as PubMed. The Reactome's and Ingenuity systems' presorted manually curated information and search tools enabled us to reliably identify and extract the pertinent entities and interactions. Identification and extraction of relevant information from appropriate primary literature is a tedious task. Although slower, use of information from the pathway resources expedited the identification step. The relevant primary literature is also populated as annotations for entities and interactions while creating the pathway (unpublished data). The efficient building and visualization of a pathway requires the use of appropriate software. We chose to assemble the pathway in CellDesigner due to its flexible graphics capabilities that facilitate a clear presentation of high granularity pathways.

DDO pathway building, which can follow a similar process, differs in that the starting point is typically a collection of genes or proteins identified in a global experiment whose relationships are not well understood. In this case, the pathway building process is used to elucidate the pathways and functional relationships shared by regulated entities.

## Pathway Analysis

Pathway analysis refers to the computational approaches used to investigate network behavior as a system. Pathway analysis can be broadly classified into two types: topological/structural network analysis and dynamical analysis.

Topological analysis of a pathway identifies the global qualitative properties of the system [6]. One approach uses classical graph theory to identify various motifs in a pathway represented as a directed graph. A motif is a group of interacting entities capable of information processing that appears repeatedly. If the graph is signed (i.e., the positive or negative regulatory effects of each interaction that may be obtained from primary literature are specified), Boolean network analysis can be used to identify the semi-quantitative features such as positive/negative feedback loops and minimal cut sets in the pathway. Feedback loops strongly affect the behavior of the system. A minimal cut set of entities is the smallest group of entities that, when disrupted, affect the particular network behavior of interest. The identification of minimal cut sets aids the assessment of the robustness of a system. Motifs, feedback loops, and minimal cut sets of a pathway connecting, for example, a receptor and a transcription factor, such as NFκB, that regulates many genes, illustrate the global properties of the system. Probabilistic graphical models approaches such as Bayesian network analysis are used to analyze and learn about the cellular networks from quantitative experimental data and to infer indirect relationships.

Dynamical analysis, a higher resolution mathematical modeling, elucidates the detailed local and certain global quantitative behaviors of the system. Dynamical analysis requires more information on the reaction parameters and initial conditions than topological approaches [6]. Deterministic dynamical analysis uses differential equations to describe reactions. Deterministic partial least square (PLS) models assume the network of pathways as a processor unit. Based on the appropriate quantitative experimental measurements of key entities in an a priori known network of pathways, PLS models can be used to predict the time-dependent cross-talk between pathways of the network under certain conditions. Another approach is stochastic modeling which uses a probabilistic representation. Deterministic models describe average behavior. Stochastic approaches are important when the absolute number of the reactant molecules in each cell is small. In this condition, the probabilistic nature of chemical reactions may affect system behavior and deterministic models may not be valid. Many software tools are available for topological and dynamical pathway analysis [7,8]. 

## Supporting Information

Table S1 A list of Frequently Used Databases, Classified Based on the Type of Information Represented, during a Biological Pathway Construction, Their Properties, and URLs A comprehensive list of databases can be found in Pathguide (http://www.pathguide.org). A, automated curation; B, both manual and automated curation; BIND, Biomolecular Interaction Network Database; BioPP, Biological Pathway Publisher; DIP, Database of Interacting Proteins; EcoCyc, Encyclopaedia of E. coli Genes and Metabolism; GNPV, Genome Network Platform Viewer; HPRD, Human Protein Reference Database; KEGG, Kyoto Encyclopedia of Genes and Genomes; M, manual curation; MetaCyc, a Metabolic Pathway database; MINT, Molecular Interation Database; MIPS, Munich Information Center for Protein Sequences; N, No; OPHID, Online Predicted Human Interaction Database; PANTHER, Protein Analysis through Evolutionary Relationship Database; PID, The Pathway Interaction Database; STKE, Signal Transduction Knowledge Environment, UNIHI, Unified Human Interactome; Y, yes.
(61 KB DOC)
