# Supplementary material for: Getting Started in Biological Pathway Construction and Analysis
Source: PLoS Comput Biol. 2008 Feb 29;4(2):e16. doi: 10.1371/journal.pcbi.0040016 (PMC2323403; doi:10.1371/journal.pcbi.0040016)
Supplement: Supplementary file 1 [file pcbi.0040016.st001.doc]

**Supporting information**

**Table S1:**

| **Database** | **Curation type** | **GO Annotation (Y/N)** | **Description** | **URL** |
| --- | --- | --- | --- | --- |
| **1. Protein-protein interactions databases** | | | | |
| BIND | M | N | 200,000 documented biomolecular interactions and complexes | <http://www.bind.ca/> |
| MINT | M | N | Experimentally verified interactions | <http://mint.bio.uniroma2.it/mint/> |
| HPRD | M | N | Elegant and comprehensive presentation of the interactions, entities and evidences | <http://www.hprd.org/> |
| MPact | B | N | Yeast interactions. A part of MIPS | <http://mips.gsf.de/genre/proj/mpact/> |
| DIP | B | Y | Experimentally determined interactions | <http://dip.doe-mci.ucla.edu/> |
| IntAct | M | Y | Database and analysis system of binary and multi-protein interactions | <http://www.ebi.ac.uk/intact> |
| PDZBase | M | N | PDZ Domain containing proteins | <http://icb.med.cornell.edu/services/pdz/start/> |
| GNPV | B | Y | Based on specific experiments and literature | <http://genomenetwork/nig.ac.jp/> |
| BioGrid | M | Y | Physical and genetic interactions | <http://thebiogrid.org/> |
| UniHi | B | Y | Comprehensive human protein interactions | <http://theoderich.fb3.mdc-berlin.de:8080/unihi/> |
| OPHID | B | Y | Combines PPI from BIND, HPRD, and MINT | <http://ophid.utoronto.ca/ophid/> |
| **2. Metabolic Pathway databases** | | | | |
| EcoCyc | B | Y | Entire genome and biochemical machinery of E. Coli | [http://ecocyc.org](http://ecocyc.org/) |
| MetaCyc | M | N | Pathways of over 165 species | [http://metacyc.org](http://metacyc.org/) |
| HumanCyc | B | N | Human metabolic pathways and the human genome | [http://humancyc.org](http://humancyc.org/) |
| BioCyc | B | N | Collection of databases for several organism | [http://biocyc.org](http://biocyc.org/) |
| **3. Signaling Pathway databases** | | | | |
| KEGG | M | Y | Comprehensive collection of pathways such as human disease, signaling, genetic information processing pathways. Links to several useful databases. | <http://www.genome.ad.jp/kegg/> |
| PANTHER | M | N | Compendium of metabolic and signaling pathways built using CellDesigner. Pathways can be downloaded in SBML format. | <http://panther.appliedbiosystems.com/> |
| Reactome | M | Y | Hierarchical layout. Extensive links to relevant databases such as NCBI, ENSEMBL, UNIPROT, HAPMAP, KEGG, CHEBI, PubMed, GO. Follows PSI-MI standards. | <http://www.reactome.org/> |
| Biomodels | M | Y | Domain experts curated biological connection maps and associated mathematical models | <http://www.ebi.ac.uk/biomodels/> |
| STKE | M | N | Repository of canonical pathways. | <http://stke.sciencemag.org/cm/> |
| Ingenuity Systems | M | Y | Commercial mammalian biological knowledgebase about genes, drugs, chemical, cellular and disease processes, and signaling and metabolic pathways. | <http://ingenuity.com/> |
| PID | M | Y | Compendium of several highly structured, assembled signaling pathways | <http://pid.nic.nih.gov/PID/> |
| BioPP | B | Y | Repository of biological pathways built using CellDesigner | <http://tsb.mssm.edu/pathwayPublisher/boradcast/> |

*Table S1:* A list of frequently used databases, classified based on the type of information represented, during a biological pathway construction, their properties and URL. A comprehensive list of databases can be found in Pathguide ([http://www.pathguide.com](http://www.pathguide.com/)). Legend: M – Manual curation, A – Automated curation, B- Both manual and automated curation; Y – Yes, N – No; BIND – Biomolecular Interaction Network Database, DIP – Database of Interacting Proteins, GNPV – Genome Network Platform Viewer, HPRD = Human Protein Reference Database, MINT – Molecular INTeration database, MIPS – Munich Information center for Protein Sequences, UNIHI – Unified Human Interactome, OPHID – Online Predicted Human Interaction Database, EcoCyc – Encyclopaedia of E. Coli Genes and Metabolism, MetaCyc – aMetabolic Pathway database, KEGG – Kyoto Encyclopedia of Genes and Genomes, PANTHER – Protein Analysis Through Evolutionary Relationship database, STKE – Signal Transduction Knowledge Environment, PID – The Pathway Interaction Database, BioPP – Biological Pathway Publisher.
